# Assessment of *Culicidae* collection methods for xenomonitoring lymphatic filariasis in malaria co-infection context in Burkina Faso

**DOI:** 10.1371/journal.pntd.0012021

**Published:** 2024-03-29

**Authors:** Sanata Coulibaly, Simon P. Sawadogo, Achille S. Nikièma, Aristide S. Hien, Rabila Bamogo, Lassane Koala, Ibrahim Sangaré, Roland W. Bougma, Benjamin Koudou, Florence Fournet, Georges A. Ouédraogo, Roch K. Dabiré

**Affiliations:** 1 Institut de Recherche en Sciences de la Santé (IRSS), Bobo-Dioulasso, Burkina Faso; 2 Université Nazi Boni, Bobo-Dioulasso, Burkina Faso; 3 Programme National de Lutte contre les Maladies Tropicales Négligées, Ministère de la Santé, Ouagadougou, Burkina Faso; 4 Centre Suisse de Recherches Scientifiques, Université Félix-Houphouët-Boigny, Abidjan, Côte d’Ivoire; 5 MIVEGEC (UM, IRD, CNRS) Montpellier, France; University of Agricultural Sciences and Veterinary Medicine Cluj-Napoca, Life Science Institute, ROMANIA

## Abstract

**Background:**

Entomological surveillance of lymphatic filariasis and malaria infections play an important role in the decision-making of national programs to control, or eliminate these both diseases. In areas where both diseases prevalence is low, a large number of mosquitoes need to be sampled to determine vectors infection rate. To do this, efficient mosquito collection methods must be used. This study is part in this framework, to assess appropriate mosquito collection methods for lymphatic filariasis xenomonitoring in a coexistence context with malaria in Burkina Faso.

**Methodology/Principal findings:**

Mosquito collections were performed between August and September 2018 in four villages (Koulpissi, Seiga, and Péribgan, Saptan), distributed in East and South-West health regions of Burkina Faso. Different collection methods were used: Human Landing Catches (HLC) executed indoor and outdoor, Window Exit-Trap, Double Net Trap (DNT) and Pyrethrum Spray Catches (PSC). Molecular analyses were performed to identify *Anopheles gambiae s*.*l*. sibling species and to detect *Wuchereria bancrofti* and *Plasmodium falciparum* infection in *Anopheles* mosquitoes. A total of 3 322 mosquitoes were collected among this, *Anopheles gambiae s*.*l*. was the vector caught in largest proportion (63.82%). *An*. *gambiae s*.*l*. sibling species molecular characterization showed that *An*. *gambiae* was the dominant specie in all villages. The Human Landing Catches (indoor and outdoor) collected the highest proportion of mosquitoes (between 61.5% and 82.79%). For the sampling vectors infected to *W*. *bancrofti* or *P*. *falciparum*, PSC, HLC and Window Exit-Trap were found the most effective collection methods.

**Conclusions/Significance:**

This study revealed that HLC indoor and outdoor remained the most effective collection method. Likewise, the results showed the probability to use Window Exit-Trap and PSC collection methods to sample *Anopheles* infected.

## Author summary

In Burkina Faso, the monitoring and evaluation scheme to assess the impact of lymphatic filariasis and malaria interventions is relied only on parasitological tests. While nowadays, the most simple and direct measure of vector borne diseases is xenomonitoring. When the both diseases prevalence is low in human population, sampling large numbers of mosquitoes is necessary for xenomonitoring. This study was undertaken in this context to assess the efficiency of four mosquito collection methods for xenomonitoring LF and malaria. Mosquito collections were performed between August and September 2018 in four villages, distributed in East and South-West health regions of Burkina Faso. Human Landing Catches (HLC), Window Exit-Trap, Double Net Trap (DNT) and Pyrethrum Spray Catches (PSC) were evaluated. The results showed that HLC remained the most effective collection method by collecting the highest number of *Anopheles* (2 388; 71.88% of total). Across the study, mosquito infection rate for *W*. *bancrofti* and *P*. *falciparum* were 0.004 and 0.13 respectively. To collect vectors infected it was found that Window Exit-Trap and PSC were efficient. In conclusion, HLC has been shown to be appropriate for collecting large number of mosquitoes. Likewise, Window Exit-Trap and PSC can be useful for malaria and lymphatic filariasis xenomonitoring.

## Introduction

Vector borne diseases are major threat to human health worldwide. According to the World Health Organization (WHO), these diseases account for about 17% of the global burden of communicable diseases and are widespread in the poorest regions of the world [1]. Malaria and lymphatic filariasis (LF) cited as one of the main mosquito-borne human diseases, exhibit a high level of morbidity and mortality in Sub-Saharan Africa. These are parasitic diseases, which *Wuchereria bancrofti* is responsible for the majority cases of LF and most malaria cases are caused by *Plasmodium falciparum* [2]. The parasites of both diseases are in majority transmitted by mosquitoes of the genus *Anopheles* in West Africa [3,4].

In Burkina Faso, nearly 80.5% of cases and an estimated 4 144 deaths due to malaria were recorded throughout the country in 2017 [5]. At the same time, the national neglected tropical diseases control program (NNTDCP) report revealed, that LF transmission is interrupted in 60 of the country’s 70 health districts. However, microfilaria prevalence remains above 1% in some health districts distributed in the Centre, Centre-East, East and South-West health regions [6].

While significant progress have been made in the purview of control and elimination of LF and malaria [7,8], through vector control and chemoprevention, an effective assessment of interventions is necessary to assess the interruption of both diseases transmission. In Burkina Faso, the monitoring and evaluation scheme to assess the impact of LF intervention is only focusing on parasitological tests by microfilariae diagnostic in human population but does not include the detection of parasite in mosquitoes [9]. With regard malaria control, in punctual studies, the scheme to assess the impact of intervention is done in one of two ways. Firstly, human blood is tested for the presence of the parasite [10]. Secondly, mosquitoes are collected and tested, either through dissection to find the parasite [11], or through the use of molecular methods to detect the DNA [12]. Nowadays, the most direct and simple timely measure of vector borne diseases transmission is through the examination of vectors, for the presence of infective stages of the parasites responsible for the infection [3]. To this effect, determine the presence of parasites in vectors, remains an option to be considered, to evaluate malaria and LF transmission after the control strategies setting up [13,14]. However, when the both diseases prevalence is low in human population, sampling large numbers of mosquitoes is necessary [15,16]. Thus, effective collection methods must be used for sampling potential vectors of pathogens.

In vector borne diseases control, several mosquito collection methods (such as: mouth aspirator catches of indoor resting mosquitoes, pyrethrum spray catches, human landing catches, attractant traps, gravid traps, entry–exit trap) used to determine the infection rate in vector population. In Burkina Faso context, sampling mosquitoes relies almost exclusively upon human landing catches [17,18]. This collection method is difficult to approve ethically, due to exposure the collectors to the bites of mosquitoes infected. So, to take these ethical issues into account, alternative traps have been designed and compared with human landing catches for monitoring these diseases [19,20]. However, no study has compared the performance of mosquito traps for malaria and LF monitoring simultaneously in the country. The present study was undertaken to assess the efficacy of four vectors collection methods for malaria and LF xenomonitoring in areas of Burkina Faso where malaria is endemic and LF persist. Specifically, to identify the different mosquito species responsible for both diseases transmission simultaneously, determine appropriate collection methods for sample large number of mosquitoes and calculate the rate of mosquitoes infected, sampled by each method.

## Methods

### Ethics statement

Ethical consent of the study was obtained from ethic committee of Institut de Recherche en Science de la Santé (Bobo-Dioulasso) under the N°A08/2014/ CEIRES.

Community engagement was obtained following meetings between a group of staff (including entomologists, anthropologists) from Institut de Recherche en Sciences de la Santé (IRSS) and the local authorities of each study village. During these meetings, IRSS staff presented the protocol and the study objectives, highlighting the strategies used to survey vector of diseases, focusing on malaria and lymphatic filariasis, and the importance of the role that villagers could play in supporting its success.

A written consent form was signed or marked with fingerprint by the participants of the HLC and DNT experiments. Only participants in collection activities had access to the consent forms. Later, the papers have stored in archives. Malaria and LF prophylaxis were provided to vectors collectors.

### Study sites

**Fig 1:**
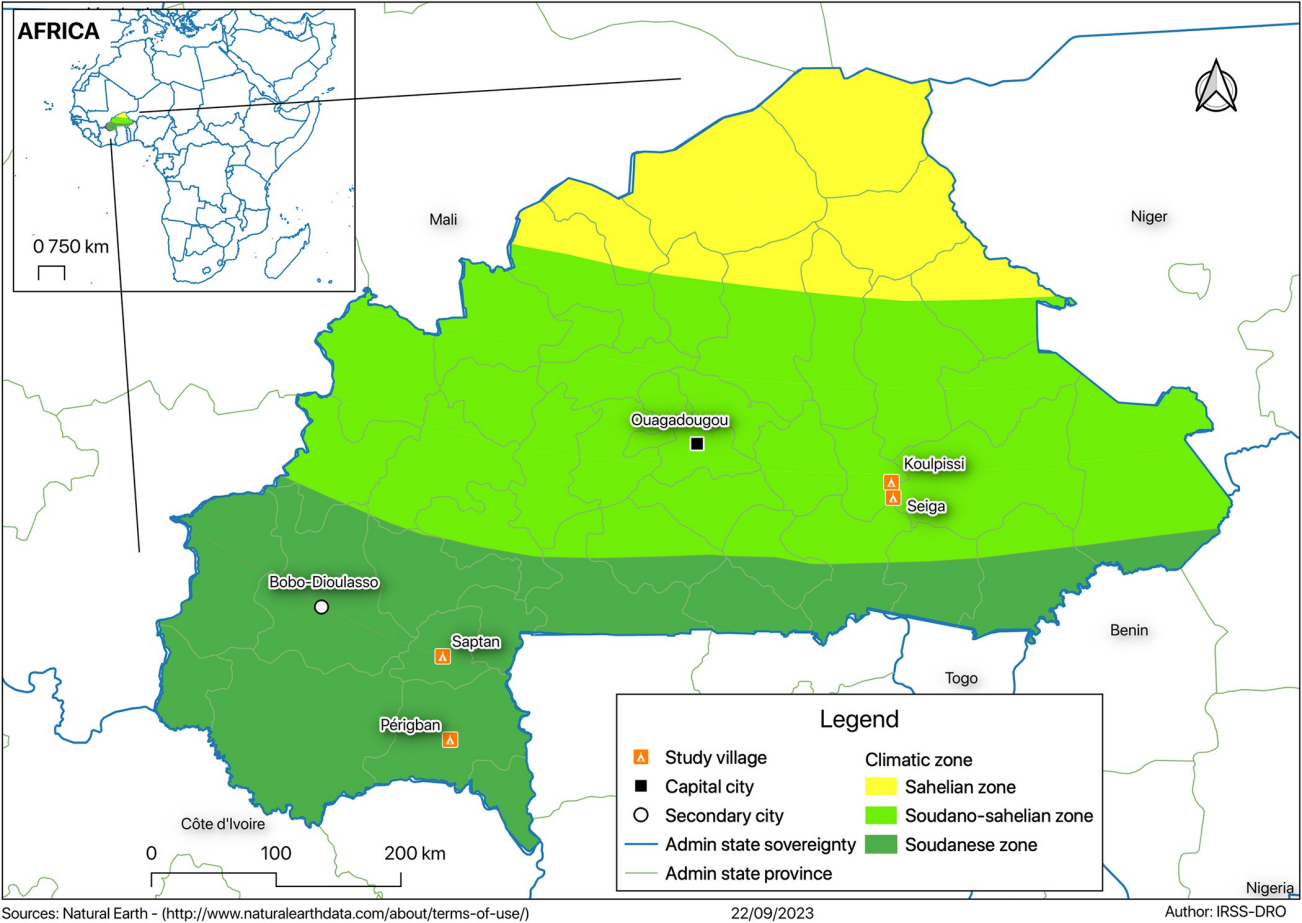
Map showing the study regions [Sources: Natural Earth-(http://www.naturalearthdata.com/about/terms-of-use)].

This study was conducted in the Fada health district (East health region) likewise in Gaoua and Diébougou health districts (South-West health region). We selected four villages for the entomological surveillance: Seiga village (-0.085971°W; 11.965555°N) and Koulpissi village (-0.097974°W; 12.078119°N) located in East health region, Saptan village (-3.404237°W; 1083015°N) and Péribgan village (-3.3387°W; 102218°N) located in South-West health region (**Fig 1**).

The East health region is located in the southeastern part of the country and bordered to south and east by Togo, Benin and Niger. Inside the country, the region is bordered by the region of the Centre-East, the region of Sahel and the region of Centre North. East health region is characterized by a sparse hydrographic networks and savanna type vegetation.

The South-West health region is in the south of Burkina Faso. The region is bordered by Côte d’Ivoire in the south and the region of Centre-West and Ghana to the east. To the north, the region of Boucle du Mouhoun and Haut Bassins border the region. To the east, the South-West health region is bordered by the Cascades Region. South-West health region is characterized by a dense hydrographic network and wooded type savannah vegetation dotted with clear forest and gallery.

### Collection methods

#### Human Landing Catches (HLC)

It is method which enables to sample mosquitoes seeking a human host for taking the blood feeding. Thus, the mosquitoes are collected when they land on exposed legs. This method is useful for assessing human-vector contact, host attractiveness, mosquito survival and infection and infectivity rates [21]. HLC is the most common method of collecting large number of mosquitoes, but it is ethically questionable due to the exposure of the collectors [15,22,23].

#### Window Exit-Trap

These traps are rectangular boxes made of a wooden or wire frame on which is stretched a mesh of braided glass fibers. On one side there is an inclined rectangular slot made of wire to allow mosquitoes to enter and on the other side there is an opening in which a cotton suction sleeve is inserted and can be closed [15,22]. Window Exit-Trap is used to monitoring some vectors species that tend to enter houses at night bite and leave the house soon after feeding without resting indoor. It provides information about exophilic versus endophilic resting behavior of vectors, physiological and biodemographic status distributions of the specimens sampled [16].

#### Double Net Trap (DNT)

It is two box nets; the inner net protects the human-bait, and the outer net is raised of the ground so that mosquitoes lured to the human-bait are collected between the nets. The nets are not treated with any insecticide [15,23]. Several study have shown that DNT collection method as effective as the Human landing catches (HLC) method in term of the number of infected mosquitoes collected but it was less effective in term of density (total number of mosquitoes collected: infected and uninfected mosquitoes)[24,25].

#### Pyrethrum Spray Catches (PSC)

This method consisted of spraying the inside of houses closed with residual aerosol insecticide very early in the morning [22].The PSC is one of the most common methods for sampling indoor-resting populations of vectors. It is used to yield information on feeding pattern survey indoor resting densities and vector species composition [16].

### Study design

This was a cross-sectional study, that was conducted in four villages (Seiga, Koulpissi, Saptan and Péribgan) distributed in East and South-West health regions of Burkina Faso. Mosquitoes were collected between August and September 2018, during the rainy season which corresponding to the period of high abundance of mosquitoes. It was preliminary study, undertaken to assess the efficacy of four mosquitoes’ collection methods for LF and malaria monitoring in areas where malaria is endemic and LF persist. In each village, two collection days were carried out and 11 households, in which mosquito samples, were conducted were chosen in random way. The households were spread distributed as follows: two households for human landing catches, whose four consenting adult volunteers are recruited and trained at each site to collect mosquitoes (one indoor and other outdoor), two households for expose DNT to outdoor, two households for the Window Exit-Trap outdoor exhibition and five households for the PSC. The households were selected conveniently: Each household in each village was numbered. After, 11 numbers were selected randomly corresponding to the 11 households. So, each household had an equal chance of being selected. To avoid the households chosen not being concentrated in the same area of the village, the village has divided into 11 areas, before choosing the households.

### Mosquito sampling

Vectors sampling by HLC and DNT were performed in four households, from 08:00 pm to 06:00 am. These two sampling methods were carried out alternately between the concessions during the two days of collection at each site. As for the Window Exit-Trap, they were kept on windows and the vectors sampling was done from 06:00 am to 09:00 am for the two consecutive collection days. PSC were done in the morning from 06:00 am to 09:00 am.

Trapped vectors were collected with aspirator and manual collection was done for mosquitoes taken in HLC and PSC. Mosquitoes were identified under a binocular magnifying glass using the identification key of Gillies and Coetzee [26].

Mosquito samples were stored on silicagel in 1.5ml tubes by species/collection method/village/period and brought to laboratory of Institut de Recherche en Science de la Santé in Bobo-Dioulasso for the molecular analyses.

### Molecular detection of *Wuchereria bancrofti* and *Plasmodium falciparum*

The head and thorax of mosquitoes from the *Anopheles* genera (*An*. *gambiae s*.*l*., *An*. *nili*, *An*. *funestus s*.*l*., *An*. *sp*) were served to genomic DNA extract. DNA was extracted with 2% Cetyl Trimethyl Ammonium Bromide (2% CTAB). Then, Sine 200X 6.1 locus protocols described by Santolamazza *et al*., [27] were used to identify the members of *An*. *gambiae* complex. To detect *W*. *bancrofti* and *P*. *falciparum* infection, DNA amplification was carried out following the procedure described by Ramzy *et al*., [28] and Echeverry *et al*., [29] respectively. Primer sequences NV-l [5′ CGTGATGGCATCAAAGTAGCG 3′ (21-mer)] and NV-2 [5′ CCCTCACTTACCATAAGACAAC 3′ (22-mer)] specific for *W*. *bancrofti* detection served to the reaction. The amplification reaction was done with final volume of 20 μL. A 2% agarose gel, stained with ethidium bromide was used for electrophoresis. The bands size expected was between 188bp and 200bp. The primers COX-IF (5′AGAACGAACGCTTTTAACGCCTG3′) and COX-IR (3′ACTTAATGGTGGATATAAAGTCCATCCwGT 5′) was used for *P*. *falciparum* detection. A reactional volume of 25 μL was used for amplification. A 1.5% agarose gel, stained with ethidium bromide was used for electrophoresis. The band size expected was ~540 bp.

### Data analysis

The statistical processing of the data was done with the software R. The interface R_Studio of R version 3.3.1 was used to perform the Chi square test (*X*^*2*^) with a probability threshold *p-value = 5%* to compare the proportion of mosquitoes sampled by collection method and by health region. The infection rates of *W*. *bancrofti* and *P*. *falciparum* in mosquitoes was estimated using the Pool Screen software 2.0 [30] with 95% confidence interval (CI) reported as the maximum likelihood. As this is a preliminary study, no gold standard has defined. The comparison was made between collection methods.

## Results

### Mosquito abundance and composition

**Fig 2:**
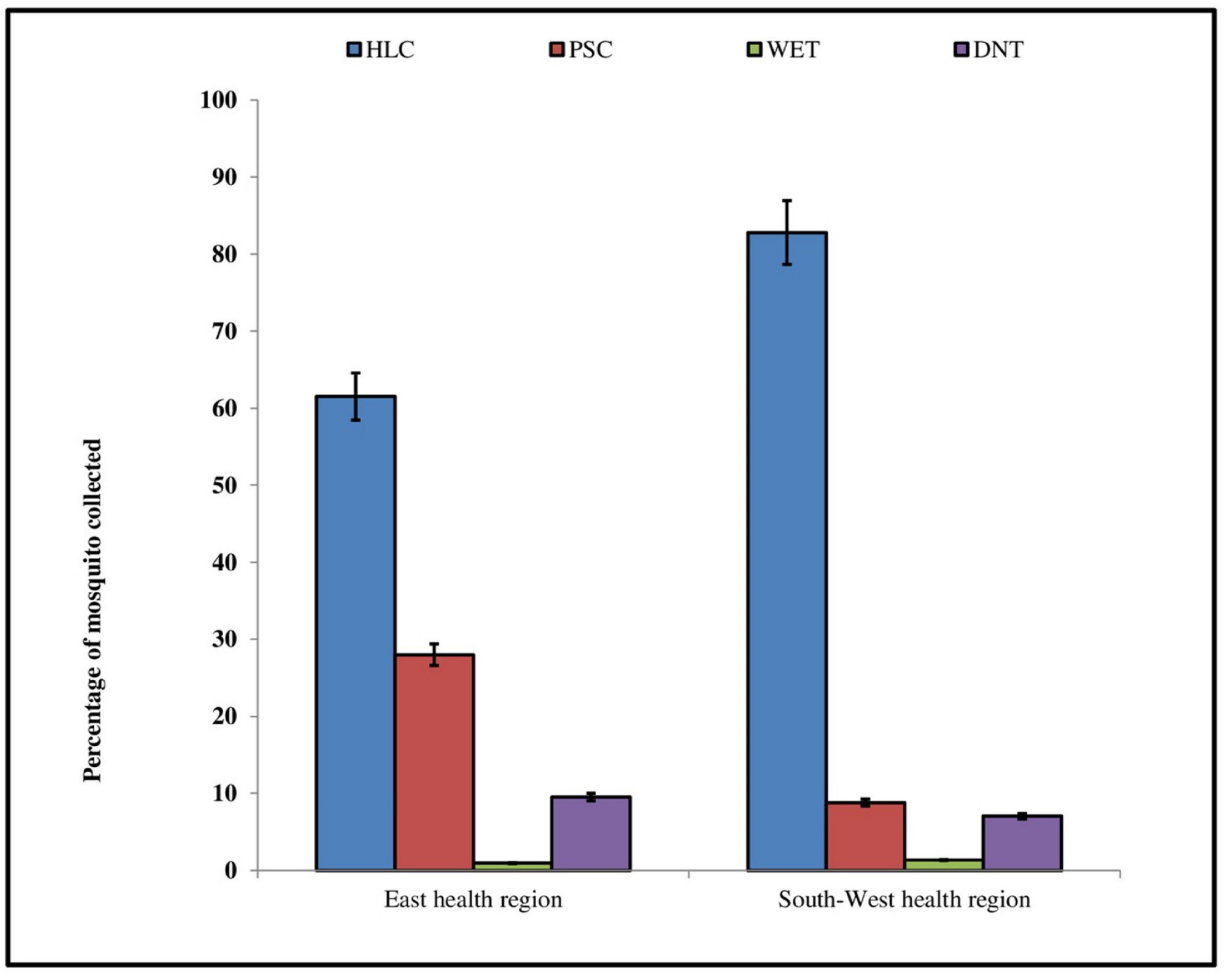
Percentages of mosquitoes (combined data) caught by sampling method in the study sites of the East and South-West health regions. (*p-value* = 2^−16^) (**HLC**: Human Landing Catches; **PSC**: Pyrethrum spray catches; **WET**: Window Exit-Trap; **DNT**: Double Net Trap).

A total of 3 322 mosquitoes were collected in the four villages distributed in the two health regions during the study period. Morphological identification of collected mosquitoes showed that 2 603 (78.35%) were filarial and malaria vectors belonging to members of the *An*. *gambiae* complex, *An*. *funestus s*.*l*. and *An*. *nili* (**Table 1**). There was difference (X^2^ = 643.19, df = 7, *p-value*< 2.2e-16) in the mosquito species composition sampled in the villages distributed in every health region. Out of the mosquitoes collected methods, Human Landing Catches collected largest number of mosquitoes 1 046 (61.5%) in the villages of East health region and 1 342 (82.79%) in the villages South-West health region (**Fig 2**).

**Table 1:** Mosquito composition by collection method in four villages distributed in two health regions of Burkina Faso (HLC: Human Landing Catches; PSC: Pyrethrum Spray Catches; WET: Window Exit-Trap; DNT: Double Net Trap).

Mosquito sub-family	Mosquito species	East health region								South-West health region								Total by mosquito species (%)
		Seiga				Koulpissi				Péribgan				Saptan				
		HLC	PSC	WET	DNT	HLC	PSC	WET	DNT	HLC	PSC	WET	DNT	HLC	PSC	WET	DNT	
*Anophelinae*	*An*. *gambiae s*.*l*.	292	185	10	5	554	208	2	9	400	36	12	3	273	93	5	33	2120 (63.82)
	*An*. *funestus s*.*l*.	0	0	0	0	0	0	0	0	8	4	0	0	2	4	0	0	18 (0.54)
	*An*. *nili*	0	0	0	0	1	0	0	0	8	0	0	0	406	0	3	5	423 (12.74)
	*An*. *sp*	0	0	0	6	3	0	0	4	2	0	0	9	12	0	0	6	42 (1.26)
*Culicinae*	*Aedes sp*	0	3	0	57	0	0	0	40	0	0	1	2	0	0	0	3	106 (3.19)
	*Culex sp*	115	48	4	15	81	33	0	26	209	2	1	42	22	4	0	11	613 (18.45)
Total by collection method		**407**	**236**	**14**	**83**	**639**	**241**	**2**	**79**	**627**	**42**	**14**	**56**	**715**	**101**	**8**	**58**	**3322 (100)**

**Fig 3:**
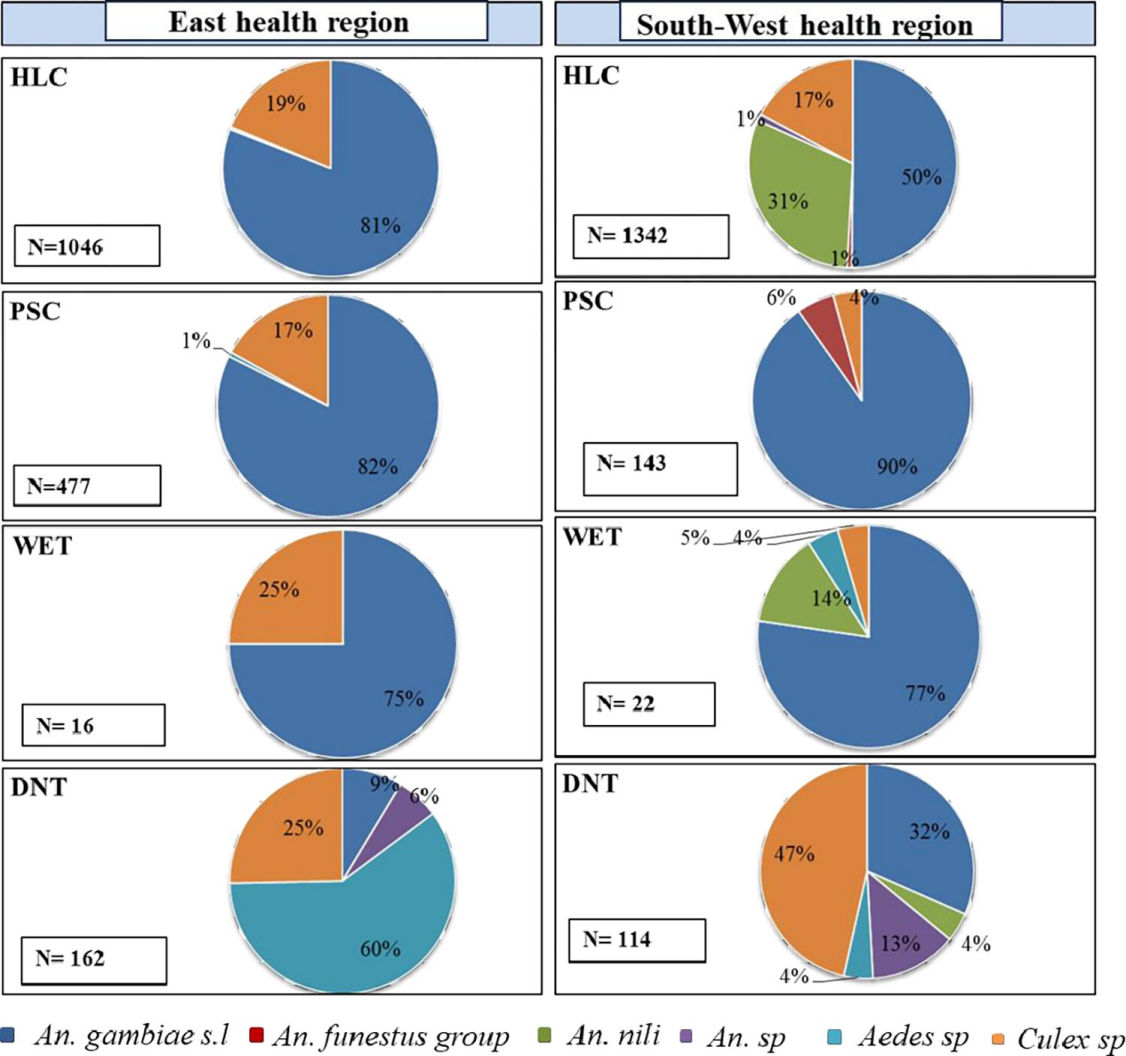
The relative percentage of the different mosquito species (combined data) caught by the four sampling methods in the study sites of the East and South-West health regions. **(**Where **‘N’** is the number of mosquitoes caught in each trap**; HLC:** Human Landing Catches**; PSC:** Pyrethrum spray catches**; WET:** Window Exit-Trap**; DNT:** Double Net Trap**)**.

In East health region villages, of the 1 701 mosquitoes collected, *An*. *gambiae s*.*l*. was collected in largest proportion using HLC (80.9%), PSC (82.4%) and Window Exit-trap. On the other hand, *Aedes sp*. (47%) was collected using DNT (**Fig 3**). No *An*. *funestus s*.*l*. has been collected in this health region villages.

In South-West health region villages, 1 621 mosquitoes were collected. *An*. *gambiae s*.*l*. were the mosquito species predominantly sampled by the HLC (50%), PSC (90%) and Window Exit-trap (77%) followed by *An*. *nili* collected in 31% and 14% by HLC and Window Exit-trap respectively. *Culex sp* (47%) was collected using DNT in this health region villages (**Fig 3**).

Only *An*. *gambiae* sibling species are molecular identified. Molecular characterization of 327 *An*. *gambiae s*.*l*. sampled in South-West health region villages has shown that 90.83% were *An*. *gambiae*, 7.95% were *An*. *coluzzii*, and 1.22% were *An*. *arabiensis*. In East health region villages, 410 *An*. *gambiae s*.*l*. were sampled and molecular analyzed; 70% were characterized as *An*. *gambiae*, 28.54% were *An*. *coluzzii* and 1.46% was *An*. *arabiensis*. These proportions did not differ significantly between the collection methods (*X*^*2*^ = 0.8, *p-value* = 0.6).

### Mosquito infection rate

As this is a preliminary study, only *Anopheles* (*An*. *gambiae s*.*l*., *An*. *funestus s*.*l*. and *An*. *nili*) unfed, sampled by every collection method, in the villages of each health region, were selected and analyzed to determine the infection rate.

Only *An*. *gambiae* sibling species have been found to be infected. From a total of 815 heads and thorax analyzed, sporozoid index was 0.13 and the microfilaria index was 0.004 in the villages of all health regions (**Table 2**). Pyrethrum spray catches and Window Exit-traps were able to collect vectors infected to *P*. *falciparum* (so the most effective collection methods to collect vectors infected to *P*. *falciparum*) in the villages of East and South-West health regions respectively. Regarding the mosquito microfilaria infection, one vector was identified in the villages of South-West health region sampled by the PSC collection method. In the villages of East health region two vectors were identified, one sampled by HLC indoor and the other by HLC outdoor. None of the analyzed mosquitoes was found to be infected.

**Table 2:** Sporozoid and microfilaria index in mosquitoes by collection method according to every health region (HLC: Human Landing Catches; PSC: Pyrethrum Spray Catches; IS: Sporozoid Index; ImF: microfilaria index; TP: total of vectors positive).

Collection method	East health region			South-West health region			General		
	Number of mosquitoes screened	IS (TP)	ImF (TP)	Number of mosquitoes screened	IS (TP)	ImF (TP)	Number of mosquitoes screened	IS (TP)	ImF (TP)
HLC Indoor	110	0.1 (11)	0.009 (1)	160	0.05 (8)	0	270	0.07 (19)	0.0037 (1)
HLC Outdoor	91	0.08 (7)	0.01 (1)	137	0.05 (7)	0	228	0.06 (14)	0.0043 (1)
PSC	201	0.28 (56)	0	89	0.17 (15)	0.01 (1)	290	0.24 (71)	0.0034 (1)
Window Exit-trap	8	0.13 (1)	0	10	0.3 (3)	0	18	0.22 (4)	0
DNT	7	0	0	2	0	0	9	0	0
Overall	417	0.18 (75)	0.005 (2)	398	0.08 (33)	0.003 (1)	815	0.13 (108)	0.004 (3)

## Discussion

The sustained success of vectors borne disease elimination depends, on a careful and comprehensive monitoring parasite infection in vector populations, to detect potential persistence and/or recrudescence after diseases control tools setting up, particularly in high-risk areas. However, several methods are used to collect infected mosquitoes [25], potential vectors of certain diseases such as LF, dengue, malaria. There is a challenge to identify efficient vector collection methods for *Anopheles* mosquitoes, the primary vectors of LF and malaria in Sub-Saharan Africa [3]. This study reports on the first evaluation of different mosquito collection methods for monitoring LF and malaria simultaneously in Burkina Faso.

In this study, *Anopheles* genus was caught in largest proportion using the HLC and PSC. *An*. *gambiae s*.*l*. was collected mainly with a significant difference in the abundance of mosquito species sampled at each health region. Indeed, the collections were carried out during the rainy season (between August and September 2018). Thus, the presence of *An*. *gambiae s*.*l*. in high proportion is probably linked to the presence of the species’ preferred breeding sites, which are mostly temporary sites. The potential reasons for HLC and PSC attractiveness between collection methods is probably the season in which collections were conducted. This can be considered as one of the limitations of our study, as it was carried out only once (during the rainy season). To this effect, rain’s presence may make some traps (DNT and Window Exit-Trap) less attractive compared to others (HLC and PSC) in the collection of *Anopheles* which are anthropophilic vectors. In the assessment of vectors collection methods, Irish *et al*., [31] found that the rainy season can be a factor limiting the attractiveness of a trap in collecting potential vectors of LF, due to the presence of alternative favorable components for their reproduction. The molecular identification of sibling species of *An*. *gambiae* complex showed that *An*. *gambiae* and *An*. *coluzzii* were the most predominant in both health regions. *An*. *gambiae* and *An*. *coluzzii* repartitions are correlated and their geographical distribution has not changed much recent years [32,33] in Burkina Faso. A recent study showed that *An*. *nili* was *W*. *bancrofti* potential vector [34] in this region. Other *Anopheles* species in particular *An*. *pharoensis*, *An*. *rufipes*, *An*. *coustani*, *An*. *flavicosta* and *An*. *pretoriensis* can be encountered [12].

In East health region, the Window Exit-trap was the least attractive trap compared to the average number of mosquitoes collected per method. The low mosquitoes proportion catch of the trap can be explained by the resting behavior of the majority vectors because Window Exit-trap is useful for sampling mosquitoes with exophilic behavior and to trap mosquitoes that leave houses for oviposition [15]. In our context vectors have probably endophage/exophage and endophilic behavior because, the majority was sampled by HLC and PSC.

Pyrethrum Spray Catches and Window Exit-trap were collection methods less attractive in mosquitoes’ sample in South-West health region. The low mosquito catch by these traps in this region could be explained by the vectors blood feeding behavior that are probably endophage and/or exophage because HLC collection methods have sampled the highest proportion of vectors.

Double net trap was found to be the effective trap in the *Aedes sp* and *Culex sp* collection during our sampling period. The highest proportion of *Culicinae* collected with this trap has been demonstrated by previous studies [31,35] in the collection of *Culex sp* potential vectors of LF in Brazil and *Aedes sp* dengue vectors in China. The mosquito species diversity collected by DNT (**Fig 3**) positioned outdoor shows that this trap is efficient to collect a broader range of vector species which have probably exophage behavior.

It was found that HLC was the collection method which enabled to sample large number of mosquitoes in all villages in both health regions. According to WHO recommendations, for LF xenomonitoring, around 10,000 mosquitoes should be analyzed. In addition, to measure the entomological inoculation rate when the intensity of malaria transmission is low in an area, sampling large number of mosquitoes is necessary. In our context, malaria and LF are transmitted by the same mosquito vectors. By also following vectors trophic behavior, we estimate that among the four collection methods, HLC is the most effective method for lymphatic filariasis monitoring as well as malaria, despite the fact that we are in endemic area of malaria.

The number of samples through analyzed PCR for the searching *P*. *falciparum* and *W*. *bancrofti* gene in this study was low (as, in the surveillance phase of LF requires processing about 10 000 mosquitoes). However, they illustrate the utility of detecting parasite DNA in mosquitoes. Thus, the infected vectors obtained from the traps, supports the evidence that these methods are useful for sampling mosquitoes and to carry out pertinent monitoring of vector borne diseases in Burkina Faso. Hence, these collection methods could be employed in monitoring vector populations which can provide valuable information to support national programs’ decision to stop mass treatment in national level. Comparison of the differences in vectors infection index between collection methods and locations were not performed. Therefore, this is a limitation as this information is important and can inform vector monitoring campaigns. No co-infection was noted in the results obtained, previous study has shown co-infection in the human population and in the mosquito in some endemic regions of the country [34].

In perspective, it would be interesting to evaluate the effectiveness of different collection methods during different times of the year to sample potential vectors, to compare differences in vectors infection index between collection methods and sites, to use PSC and Window Exit-trap in addition to HLC for xenomonitoring, to examine the use of other collection methods such as *Anopheles* gravid trap (AGT) used in Ghana for the collection vectors potential of *P*. *falciparum* and *W*. *bancrofti* [22].

## Conclusion

In both study regions, the traditional collection method human landing catches, was a very efficient collection method compared with the other traps, so particularly in the Eastern health region where the highest mosquitoes were found resting indoors. While the PSC, Window Exit-trap and HLC showed efficiency in trapping infected mosquitoes, there are limitations in relation to the fact that the collection was done only once and during the rainy season. Thus, as these are preliminary results, more in-depth studies are undergoing to guide the two national programs decision for best integrated management for malaria and lymphatic filariasis control.
